# PDGF is a potent initiator of bone formation in a tissue engineered model of pathological ossification

**DOI:** 10.1002/term.2320

**Published:** 2017-03-20

**Authors:** Owen G. Davies, Liam M. Grover, Mark P. Lewis, Yang Liu

**Affiliations:** ^1^ Centre for Biological Engineering, Wolfson School of Mechanical and Manufacturing Engineering Loughborough University Loughborough UK; ^2^ School of Chemical Engineering University of Birmingham Birmingham UK; ^3^ School of Sport, Exercise and Health Sciences, National Centre for Sport and Exercise Medicine (NCSEM), Arthritis Research UK Centre for Sport, Exercise and Osteoarthritis Loughborough University Loughborough UK

**Keywords:** bone, PDGF, skeletal muscle, inflammation, osteoprogenitor, heterotopic ossification

## Abstract

Heterotopic ossification (HO) is a debilitating condition defined by the rapid formation of bone in soft tissues. What makes HO fascinating is first the rate at which bone is deposited, and second the fact that this bone is structurally and compositionally similar to that of a healthy adult. If the mechanisms governing HO are understood, they have the potential to be exploited for the development of potent osteoinductive therapies. With this aim, a tissue‐engineered skeletal muscle was used model to better understand the role of inflammation on this debilitating phenomenon. It was shown that myoblasts could be divided into two distinct populations: myogenic cells and undifferentiated ‘reserve’ cells. Gene expression analysis of myogenic and osteoregulatory markers confirmed that ‘reserve’ cells were primed for osteogenic differentiation but had a reduced capacity for myogenesis. Osteogenic differentiation was significantly enhanced in the presence of platelet‐derived growth factor (PDGF)‐BB and bone morphogenetic protein 2 (BMP2), and correlated with conversion to a Sca‐1^+^/CD73^+^ phenotype. Alizarin red staining showed that PDGF‐BB promoted significantly more mineral deposition than BMP2. Finally, it was shown that PDGF‐induced mineralization was blocked in the presence of the pro‐inflammatory cytokines tumour necrosis factor‐α and interleukin 1. In conclusion, the present study identified that PDGF‐BB is a potent osteoinductive factor in a model of tissue‐engineered skeletal muscle, and that the osteogenic capacity of this protein was modulated in the presence of pro‐inflammatory cytokines. These findings reveal a possible mechanism by which HO develops following trauma. Importantly, these findings have implications for the induction and control of bone formation for regenerative medicine. © 2016 The Authors Journal of Tissue Engineering and Regenerative Medicine Published by John Wiley & Sons Ltd.

## 1 Introduction

Heterotopic ossification (HO) is a debilitating condition defined by the *de novo* formation of bone within non‐osseous soft tissues. The formation of bone at atypical sites can have serious consequences such as neurovascular entrapment, and has a significant impact on an individual's quality of life (Colachis *et al*., 2004). Acquired HO is associated with severe trauma that can result as a consequence of injuries, burns or changes in local biomechanics following reparative surgeries (Davies *et al*., 2015). Despite continued efforts to determine the cellular and molecular events governing HO, the underlying mechanisms remain elusive. One of the body's initial responses to trauma is to mount a hyper‐inflammatory response, via the recruitment of cells through the local vasculature (Lenz *et al*., 2007). Previous studies have identified an association between inflammatory cytokine and chemokine expression and heterotopic ossification (Evans *et al*., 2012), and it has been suggested that certain combinations of these proteins may be used to predict the onset of HO (Forsberg *et al*., 2014). However, at present, the exact relationship between post‐trauma inflammation and HO remains unknown.

To date, several studies have attempted to profile cytokines present within the local wound microenvironment and circulation following traumatic insult. These studies have identified systemic and local upregulation of inflammatory cytokines such as interleukins (IL) and tumour necrosis factor alpha (TNFα) (Evans *et al*., 2012; Forsberg *et al*., 2014), as well as growth factors such as transforming growth factor beta (TGFβ), insulin‐like growth factor (IGF) and platelet‐derived growth factor (PDGF) (Pasinetti *et al*., 1993; Kim *et al*., 1996). Evidence identifying a possible link between the presence of a hyper‐inflammatory environment and the development of ectopic ossification was provided when mesenchymal stem cell cultures were shown to undergo osteogenic differentiation following exposure to the wound effluent of blast‐injured mice (Cadosch *et al*., 2010). Evidence of an association between post‐trauma inflammation and ectopic bone formation has also been acquired in skeletal muscle cultures, where exposure to serum derived from traumatic brain injury (TBI) patients was shown to lead to increased local proliferation and subsequent tissue mineralization (Cadosch *et al*., 2010).

**Figure 1:**
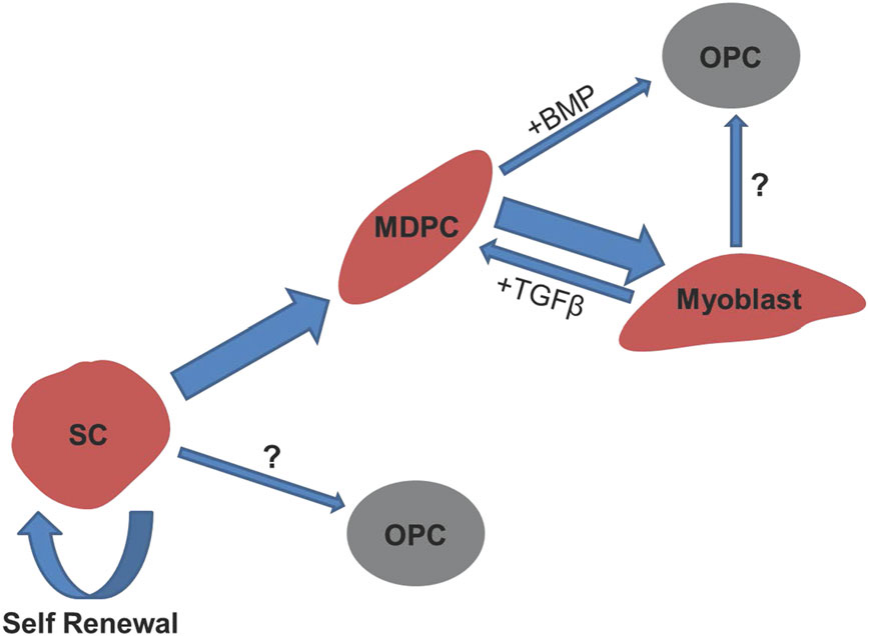
Hypothetical representation of how osteogenic progenitors may be derived from satellite cells activated following skeletal muscle trauma. SC, satellite cell; MDPC, muscle‐derived precursor cell; OPC, osteogenic progenitor cell. Arrow size represents the relative proportion of cells likely to differentiate down a given lineage

Skeletal muscle represents one of the primary sites of HO. It contains several populations of cells with a capacity for osteogenic differentiation. However, the contribution of these endogenous cells to HO remains largely unknown. Possible contributors include satellite cells and a number of mesenchymal progenitor cells such as interstitial cells (PDGFRα^+^/Sca‐1^+^), muscle‐derived haematopoietic stem cells (CD45^+^/Sca‐1^+^) and CD31^–^/CD45^–^ side population mesenchymal stem cells (Peault *et al*., 2007). However, osteogenic differentiation within skeletal muscle is not restricted to multipotent progenitors and resident stem cells. A capacity for osteogenic differentiation has also been observed within the resident myogenic cell population (Liu *et al*., 2009), with rat (L6 cells) and mouse (C2C12 cells) skeletal muscle cells shown to undergo osteogenic differentiation after treatment with osteogenic inducers, such as BMP2 or IL17 (Katagiri *et al*., 1994; Kocic *et al*., 2012). Myoblast ossification has also been shown to occur via myoblast dedifferentiation to an uncommitted and multipotent progenitor. Such reversion has been observed in the presence of TGFβ (Mu and Li, 2011), where incubation of primary myoblasts with a transient and small concentration of this cytokine has promoted the expression of primitive skeletal muscle markers, Pax7 and Sca‐1. To date, several TGFβ isoforms have been identified in both immature and mature ectopic ossifications, with this cytokine likely to play a temporal role in HO (Suutre *et al*., 2009). This has led us to hypothesize that the presence of biochemical factors within the wound environment during skeletal muscle repair and regeneration could lead to the misdirected differentiation of endogenous skeletal muscle progenitors to an osteogenic phenotype (Figure 1).

The mechanism by which ectopic ossification occurs is a largely unknown phenomenon, primarily owing to the overwhelming complexity of the body's post‐trauma response. In order to gain a better understanding of the complex processes governing HO a tissue engineered skeletal muscle model has been utilised, in a manner analogous to muscle damage and regeneration, to analyse the influence of inflammatory cytokines on heterotopic ossification. Inflammatory factors were selected based on proteomic studies of serum and wound effluent following trauma (Pasinetti *et al*., 1993; Kim *et al*., 1996; Evans *et al*., 2012). It is believed that this currently represents the only tissue‐engineered model of HO, and it is anticipated that it will complement current animal models to better understand this complex and debilitating condition.

## 2 Materials and methods

### 2.1 Cell culture

In the present study, C2C12 immortalized murine myoblasts (CRL‐1772; ATCC, Rockville, MD, USA) were used between passages six and nine. Cells were grown in T175 flasks in a humidified 5% CO_2_ atmosphere at 37°C in growth medium composed of high glucose Dulbecco's Modified Eagle Medium (DMEM) (Sigma‐Aldrich, Cambridge, UK) containing 20% fetal bovine serum (FBS) (PAA Laboratories Ltd., Yeovil, UK) and 1% penicillin/streptomycin (Thermo Fisher Scientific, Paisley, UK).

### 2.2 Preparation of tissue‐engineered skeletal muscle

To produce tethered collagen constructs, 1.3 ml type I rat‐tail collagen (in 0.1 m acetic acid, protein concentration of 2.035 mg/ml) (First Link Ltd., Wolverhampton, UK) was added to 0.15 ml 10× Minimum Essential Medium (MEM) (Gibco) and mixed thoroughly. The acidic solution was neutralized in a drop‐wise manner using 5 m and 1 m sodium hydroxide (VWR, Lutterworth, UK) until a colour change (yellow to pink) was observed. The neutralized collagen solution was mixed with a suspension of 0.15 ml growth medium containing 6 × 10^6^ C2C12 cells. A 1.5 ml aliquot of complete collagen/cell solution was pipetted into a rectangular well culture plate (Cat#267062, 37.6 × 27.9 mm; Nunc, Rochester, USA), which had been converted to 2.5 × 7.3 × 1.5 cm dimensions using an impermeable strip of silicone elastomer mould (Univar, Bradford, UK). Longitudinal lines of uniaxial tension were created through the collagen gel by tethering it at each end with bespoke polyethylene mesh flotation bars (Darice Inc., Strongsville, OH, USA) that had been bound together using stainless steel orthodontic wire.

Culture plates were placed in a humidified incubator at 37°C for 30 min to allow gelation of the collagen matrix. Once set, the construct was detached from the sides and base of the culture dish using a sterile needle, and the silicone moulds removed. The constructs were cultured in 5 ml growth medium for a period of 4 days, with the medium changed twice daily. To induce myoblast fusion, at day 5 growth medium was replaced with myogenic medium, which was composed of high‐glucose DMEM containing 2% horse serum (Sigma) and 1% penicillin/streptomycin. Constructs were cultured in myogenic medium for a period of 10 days, with daily medium changes.

### 2.3 Fluorescence and confocal microscopy

Tissue‐engineered skeletal muscle constructs were washed with 1% Tris‐buffered saline (TBS) to remove culture medium and fixed using methanol–acetone (1:1) (Sigma) for 30 min at room temperature. Fixative was removed and constructs washed twice with 1% TBS. A blocking solution composed of 5% goat serum (Gibco) and 0.2% triton‐X (Sigma) was made up in 1% TBS. Each construct was immersed in 500 μl blocking solution for 2 h at room temperature. Blocking solution was replaced with 500 μl primary antibody solution [1:200 rabbit anti‐desmin (ab86083; Abcam, Cambridge, UK), 2% goat serum, 0.2% triton‐X, made up in TBS] and each construct was incubated at room temperature overnight. Primary antibody solution was removed and each construct washed extensively with 1% TBS. A secondary antibody solution was prepared containing 1:200 goat anti‐rabbit tetramethylrhodamine (TRITC, ab6718; Abcam), 2% goat serum, 0.2% triton‐x made up in 1% TBS. Constructs were incubated in the dark at room temperature with secondary antibody solution for 2 h. The solution was removed and the constructs washed extensively with 1% TBS. To visualise nuclei each construct was incubated with 4′,6‐diamidino‐2‐phenylindole (DAPI; Sigma), 1:10 000 dilution in 1% TBS, for 10 min at room temperature. Constructs were washed using 1% TBS and mounted using DPX mounting medium (Sigma). Aligned myotubes were visualized using both a Leica DM2500 fluorescence microscope (Leica, Cambridge, UK) and a Zeiss LSM780 confocal microscope (Zeiss, Oberkochen, Germany). Confocal image reconstruction and scale measurements were performed using Zen software (Zeiss). Scale measurements for images obtained using the fluorescence microscope were generated using ImageJ software.

### 2.4 Factorial design of experiment

The independent and combinatorial effects of growth factors and cytokines identified in serum/effluent were investigated following trauma on tissue engineered skeletal muscle. A total of 16 combinations of growth/inflammatory factors were examined. Eight factors were examined in a two‐level (present or not) full factorial design: IL1β (0.02 ng/ml; Barksby *et al*., 2006), IL6 (10 ng/ml; Nasi *et al*., 2015), PDGF‐BB (2 ng/ml; Zhao *et al*., 2016), TNFα (1 ng/ml; Lam *et al*., 2000), TGFβ (1 ng/ml; Pfeilschifter *et al*., 1990), BMP2 (300 ng/ml; Katagiri *et al*., 1994), basic fibroblast growth factor (bFGF) (1 ng/ml; Coipeau *et al*., 2009) and IGF‐1 (2 ng/ml; Blumenfeld *et al*., 2000). Concentrations used were consistent with those documented throughout the literature. After 14 days' culture, mature tissue‐engineered skeletal muscle was exposed to each treatment. All proteins were dissolved in 7 ml myogenic medium and added to tissue‐engineered skeletal muscle cultures for 3 days. All experiments were designed and analysed using MiniTAB statistical software version 16 (MiniTAB Inc., State College, PA, USA). All recombinant proteins were purchased from Peprotech (Rocky Hill, NJ, USA).

### 2.5 Isolation of undifferentiated C2C12 myoblasts from tethered collagen gels

**Figure 2:**
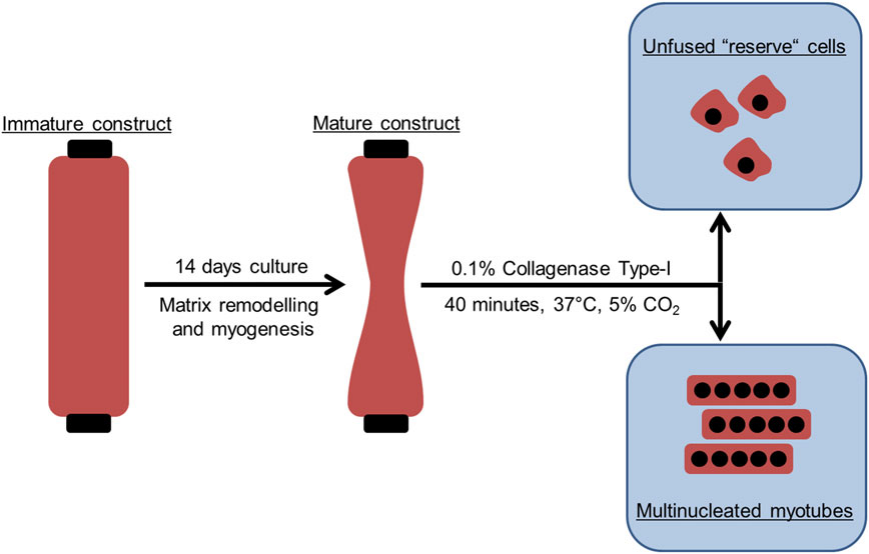
Schematic representation of the method used for isolating undifferentiated ‘reserve’ cells from tissue‐engineered skeletal muscle constructs

To isolate cells from the three‐dimensional (3D) collagen matrix for flow cytometry profiling and quantitative real‐time reverse transcription polymerase chain reaction (qRT‐RT‐PCR) analysis, the gels were placed in 50 ml centrifuge tubes (Fisher Scientific, Loughborough, UK) containing 0.1% type I collagenase (Sigma) made up in 4 ml unsupplemented DMEM (Figure 2). Based on data generated from preliminary studies, the gels were digested for a period of 40 min at 37°C and 5% CO_2_. When the gels had been fully digested the solution was neutralized through the addition of an equal volume of growth medium (20% FBS). The total cell population was collected by pelleting at 2000 ***g*** for 5 min. The heterogeneous mixture of myogenic and undifferentiated cells was seeded in 6‐well plates (Nunc). Myotubes did not adhere to tissue culture polystyrene when plated. Fragments of non‐adherent myotubes were removed after 24 h of culture and the medium renewed. Undifferentiated C2C12s were selected using this method. All images were captured using a Leica DMIL LED microscope (Leica).

### 2.6 Osteogenic differentiation of untreated ‘reserve’ cells

High‐glucose DMEM was supplemented with 50 μg/ml ascorbic acid (Sigma) and 10 mm β‐glycerophosphate (Sigma). Osteogenic medium was applied to undifferentiated cells isolated from tissue‐engineered skeletal muscle and C2C12 controls. All cells were seeded at a density of 1.5 × 10^4^ cells/cm^2^ and cultured in osteogenic medium for a period of 14 days. Medium was changed every 3 days.

### 2.7 Osteogenic differentiation of ‘reserve’ cells exposed to growth/inflammatory factors

‘Reserve’ C2C12s that had been exposed to recombinant growth factors and cytokines in 3D were isolated as previously described and seeded at a density of 1.5 × 10^4^ cells/cm^2^ and cultured in the presence of myogenic medium at 37°C and 5% CO_2_. Note that these cells were not exposed to osteogenic differentiation medium. ‘Reserve’ C2C12s that had not been exposed to inflammatory/growth factors and C2C12s previously cultured in two dimensions were used as controls. All cells were cultured at 37°C and 5% CO_2_ for 48 h.

### 2.8 Alizarin red staining and quantification

Each culture was fixed using methanol–acetone (1:1) for 20 min at room temperature. Fixed cultures were stained at room temperature for a period of 20 min using 40 mm Alizarin red (AR; Sigma). To ensure even distribution of AR stain cultures were placed on a Max400 orbital shaker (Thermo Scientific), which provided gentle agitation. Unbound stain was eluted by successive washes in phosphate‐buffered saline (PBS). Images of AR stained cultures were captured using a DMIL LED microscope (Leica). Bound AR stain was eluted using 10% (*v*/v) acetic acid (Fisher Scientific) and quantified using a Varioskan Flash plate reader (Thermo Scientific) at a wavelength of 405 nm.

### 2.9 Flow cytometry

All analysis and cell sorting was performed using a BD FACSJazz™ instrument (Becton Dickinson Immunocytometry Systems, Oxford, UK) and analysed using FlowJo version 10 software (De Novo Software, Los Angeles, CA, USA). In preparation for fluorescence‐activated cell sorting (FACS) analysis unfused cells were counted (1:1 Trypan blue) using a haemocytometer and resuspended in ice‐cold sterile PBS containing 1% FBS at a density of 1 × 10^6^ cells/ml. Conjugated primary antibodies raised against Sca‐1 (APC conjugated, 17‐5981; eBioscience Ltd., San Diego, CA, USA) and CD73 (PE‐Cy7 conjugated, 25‐0731; eBioscience Ltd., San Diego, CA, USA) were added in combination and cultures incubated in the dark at room temperature for 30 min. To remove unbound stain, cells were resuspended in sterile PBS and centrifuged at 400 ***g*** for 5 min. This washing step was repeated three times. For cell selection the FACSJazz™ instrument was equipped with an 85 μm nozzle, and cells were processed at a low sort rate using a pressure of 45 psi (310KPa). Cells were acquired and gated using forward scatter (FSC) and side scatter (SSC) parameters to exclude cell debris and aggregates. Propidium iodide (PI) staining (00‐6990‐50e; Biosciences) was used to identify and exclude non‐viable cells. These cells were excluded before analysis.

### 2.10 Quantitative real‐time PCR

Briefly, total RNA was extracted by resuspending cells in 500 μl TRI reagent (Invitrogen, Paisley, UK), according to the manufacturer's instructions. RNA was phase‐separated in chloroform and precipitated using isopropanol (both Sigma). Total RNA concentration was determined for each sample using a NanoDrop 2000 spectrophotometer (Thermo Scientific). All primers were predesigned and purchased from Sigma‐Aldrich (Table 1) (Kicqstart primers; Sigma, Irvine, UK). Quantitative real‐time RT‐PCR was performed using Quantifast™ SYBR® Green RT‐PCR one‐step kit on a ViiA™ 7 Real‐Time PCR system (Life Technologies). Parameters used were: hold 50°C for 10°min, 95°C for 5 min, 95°C for 10 s, 60°C for 30 s. Upon completion, dissociation/melting curve analyses were performed to exclude non‐specific amplifications and primer dimers. The PCR data were normalized to the reference gene ribosomal protein IIB (*RPIIB*). Relative gene expression levels were calculated from the cycle threshold (C_t_) value using the comparative Ct equation (ΔΔC_t_) or Livak method, where relative gene expression is calculated as 2^–ΔΔCt^.

**Table 1:** A list of primers used for quantitative real‐time reverse‐transcription polymerase chain reaction gene expression analysis

Gene	Sequence (5′–3′)	Accession Number
RPIIB	F‐GGTCAGAAGGGAACTTGTGGTAT	NM_153798.2
	R‐GCATCATTAAATGGAGTAGGCGTC	
MyoD1	F‐CGCTCCAACTGCTCTGATGGCA	NM_010866.2
	R‐TGCTGCTGCAGTCGATCTCTCA	
Myog	F‐GCAATGCACTGGAGTTCG	NM_031189.2
	R‐ACGATGGACGTAAGGGAGTG	
Pax7	F‐GACAAGAAAGAAGAAGATGGC	NM_011039.2
	R‐GTTCTGATTCCACATCTGAG	
Runx2	F‐ACAAGGACAGAGTCAGATTAC	NM_1145920.2
	R‐CAGTGTCATCATCTGAAATACG	
Sp7	F‐TGCTTGAGGAAGAAGCTC	NM_130458.3
	R‐CTTCTTTGTGCCTCCTTTC	
Ptch1	F‐CAACCAAACCTCTTGATGTG	NM_008957.2
	R‐CTCAAAGAGACCTAAGAGGTAG	

All KiCqStart® SYBR Green primers were purchased from Sigma‐Aldrich, Irvine, UK.

### 2.11 Data analysis and statistics

ANOVA was performed using SPSS 10.0 for Windows (SPSS Inc., Chicago, IL, USA). Principal component analysis was performed using MiniTAB® v. 16 (Minitab Inc., State College, PA, USA) statistical software to analyse main and interactive effects. This programme applied linear regression to analyse main effects and two‐way interactions. *P*‐values less than 0.05 were considered statistically significant.

## 3 Results

**Figure 3:**
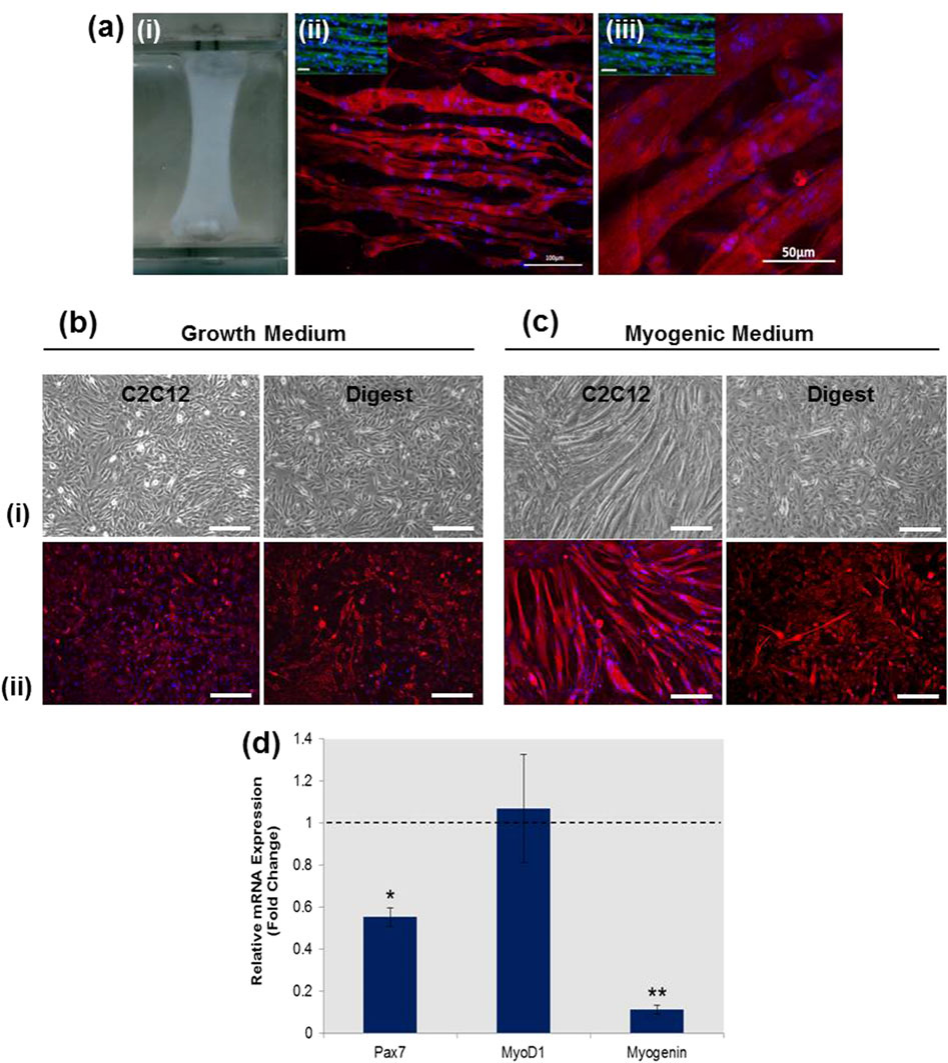
Tissue‐engineered skeletal muscle constructs contained populations of myogenic and undifferentiated ‘reserve’ C2C12s. (a) A three‐dimensional tissue‐engineered collagen gel (i) promoting the fusion and alignment of C2C12 myoblasts, as shown by fluorescence (ii) and confocal (iii) images. (b) Photomicrographs (i) of cells released from collagenase type‐I digested collagen gels, identifying a population of undifferentiated ‘reserve’ cells that did not contribute to the formation of myotubes but exhibited desmin positivity (ii). (c) Three‐day culture of these ‘reserve’ cells in a two‐dimensional environment in the presence of myogenic medium promoted only limited myogenic differentiation when compared with C2C12 controls. (d) quantitative real‐time reverse‐transcription polymerase chain reaction analysis of relative fold‐change in mRNA expression for ‘reserve’ cells showed significant reductions in the expression of Pax7 (*p* < 0.05) and myogenin (*p* < 0.005) when compared with C2C12s cultured in two dimensions. Bar: 100 μm. *p < 0.05, **p < 0.005. PCR data represented as mean ± standard deviation (*n* = 3)

The C2C12 myoblasts cultured in tissue engineered collagen gels could be separated into distinct myogenic and non‐myogenic ‘reserve’ cell populations. Myogenic cells aligned and fused to form myotubes when cultured in a 3D environment in the presence of myogenic medium (Figure 3a). ‘Reserve’ cells did not form myotubes when cultured in three dimensions (Figure 3b) and displayed limited myogenic differentiation when isolated and culture in two dimensions, even in the presence of myogenic medium (Figure 3c). Both myogenic and ‘reserve’ cell populations stained positively for the intermediate filament protein desmin (Figure 3bII,cII), which is routinely used to identify cells of a myoblast lineage. Quantitative real‐time PCR analysis was used to compare the myogenic gene expression profile of ‘reserve’ cells with C2C12 controls that had not undergone 3D selection. ‘Reserve’ cells displayed no significant change in MyoD1 expression. However, these cells exhibited a significantly reduced expression of the satellite cell marker Pax7, and the myoblast fusion marker, Myogenin (Figure 3d).

**Figure 4:**
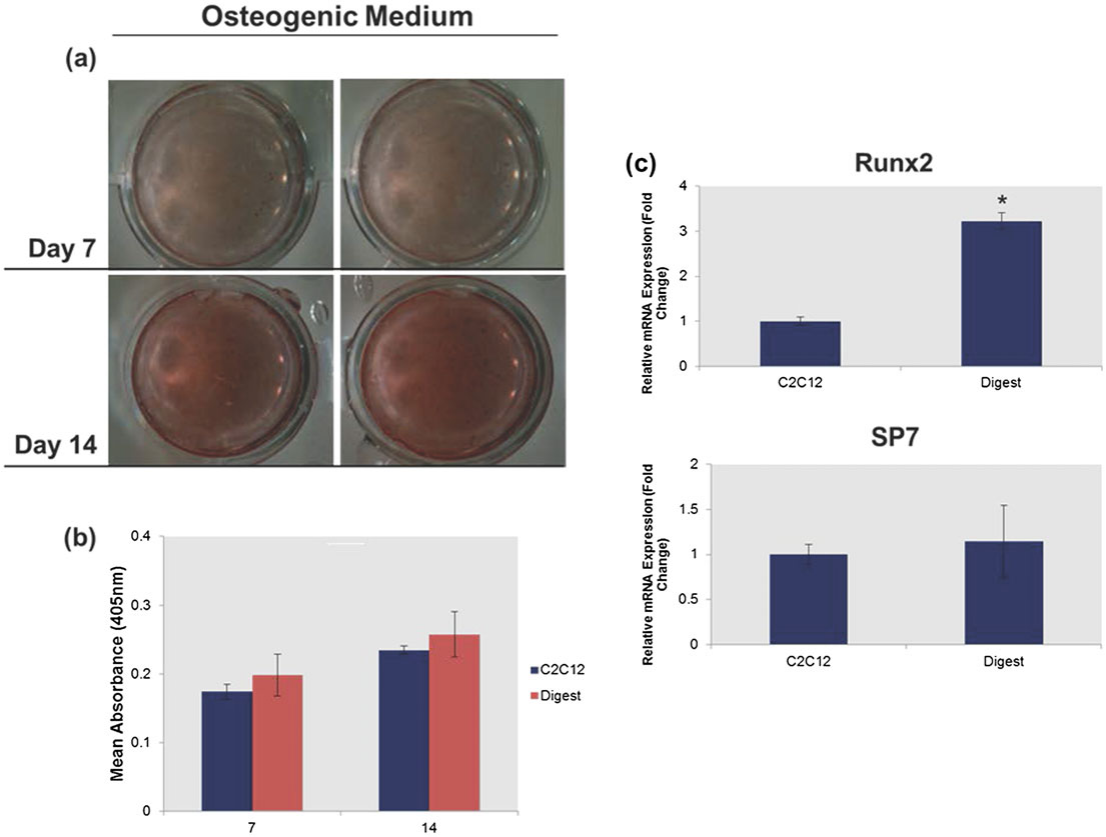
Determining the osteogenic capacity of ‘reserve’ cells. Photomicrographs and accompanying Alizarin red staining and quantification showed that ‘reserve’ cells cultured in the presence of osteogenic medium exhibited no (a) observable or (b) quantifiable differences in the formation of calcified nodules after 7 days and 14 days in culture. (c) quantitative real‐time reverse‐transcription polymerase chain reaction analysis identified a significantly (*p* < 0.05) higher expression of the master osteogenic regulator gene Runx2 in the ‘reserve’ cell population when compared with C2C12 controls after 3 days of culture. Bar: 100 μm. *p < 0.05; polymerase chain reaction and Alizarin red quantification data are represented as mean ± standard deviation (*n* = 3)

It was next determined whether undifferentiated ‘reserve’ cells isolated from tissue‐engineered muscle were capable of producing a calcified matrix when cultured in the presence of osteogenic medium for a period of 14 days. Alizarin red staining was used to identify the presence of calcified nodules and the results compared with a C2C12 population that had not undergone 3D selection. The AR staining and quantification showed no significant differences in mineralization between ‘reserve’ and control C2C12 cultures (Figure 4a,b). Gene expression analysis showed that ‘reserve’ cells exhibited a significantly increased expression in the master osteogenic regulator, Runx2 (Figure 4c) and it is believed that this was indicative of cells primed for osteogenic differentiation. However, the addition of exogenous factors is likely required to induce mineralization. No comparative increase was observed for the expression of SP7 between ‘reserve’ cells and C2C12 controls (Figure 4c).

**Figure 5:**
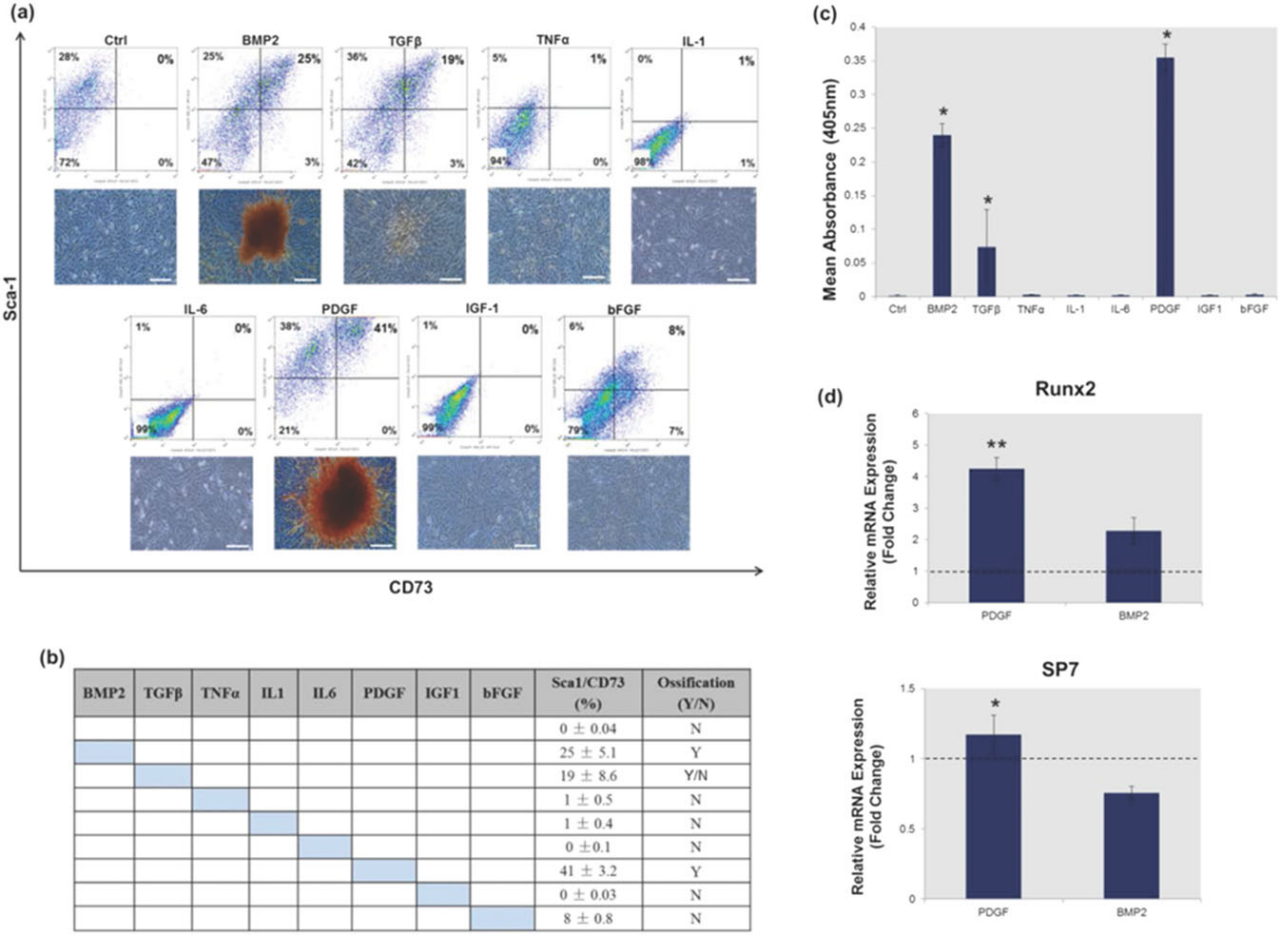
Mineralization can be induced in the undifferentiated ‘reserve’ cell population. (a) Flow cytometry profiling and accompanying Alizarin red staining identified a relationship between the presence of Sca‐1^+^/CD73^+^ cells and the formation of calcified nodules. (b) Exposure to tumour necrosis factor (TNF)α, interleukin (IL)1, IL6, insulin‐like growth factor (IGF)‐1 and basic fibroblast growth factor (bFGF) caused no significant increase in the number of Sca‐1^+^/CD73^+^ cells, and no corresponding mineralization was observed. (c) Alizarin red quantification showed that mineralization in the isolated ‘reserve’ cell population was significantly (*p* < 0.05) enhanced when skeletal muscle constructs were exposed to platelet derived growth factor (PDGF)‐BB or bone morphogenetic protein 2 (BMP2), respectively, when compared with untreated controls. (d) Gene expression analysis showed that PDGF‐BB promoted a significant (*p* < 0.05) increase in Runx2 and SP7 when compared with BMP2, which reduced SP7 expression when compared with C2C12 controls. All quantitative real‐time reverse‐transcription polymerase chain reaction analysis was performed at day 3 and normalized to untreated three‐dimensional controls using the ΔΔCT (Livak) method, indicated by the dashed lines. Coloured cells signify the addition of a growth/inflammatory factor. Bar: 100 μm. *p < 0.05, **p < 0.005; flow cytometry, polymerase chain reaction and Alizarin red quantification data are represented as mean ± standard deviation (*n* = 3)

The presence of a hyper‐inflammatory environment following trauma is well established. Hence, whether factors upregulated at the wound site following trauma could promote mineralization within the isolated ‘reserve’ cell population was evaluated next. The results demonstrated that exposure of 3D muscle constructs to BMP2 led to mineral deposition within the ‘reserve’ population following their isolation and expansion in two dimensions (Figure 5a,b). Flow cytometry profiling identified that mineralization within the ‘reserve’ population correlated with a significant increase in the proportion of cells displaying a Sca‐1^+^/CD73^+^ phenotype (Figure 5a,b). The proportion of Sca‐1^+^/CD73^+^ cells in cultures exposed to TNFα (1%), IL1 (1%), IL6 (0%), IGF‐1 (0%) and bFGF (8%) was negligible, and no subsequent mineralization was observed (Figure 5a–c). Exposure to TGF‐β caused a 19% increase in Sca‐1^+^/CD73^+^ phenotype and small sporadic areas of mineral deposition. No significant changes were observed in the expression of mesenchymal stem cell (MSC) surface markers CD90, CD105 or CD140 for any of the treatments analysed (data not included). Exposure to PDGF‐BB promoted significantly more mineralization when compared with BMP2 treatment (Figure 5c). A qRT‐RT‐PCR analysis of osteogenic regulator genes identified a significantly higher expression of Runx2 and SP7 in PDGF‐BB cultures compared with BMP2 cultures (Figure 5d).

**Figure 6:**
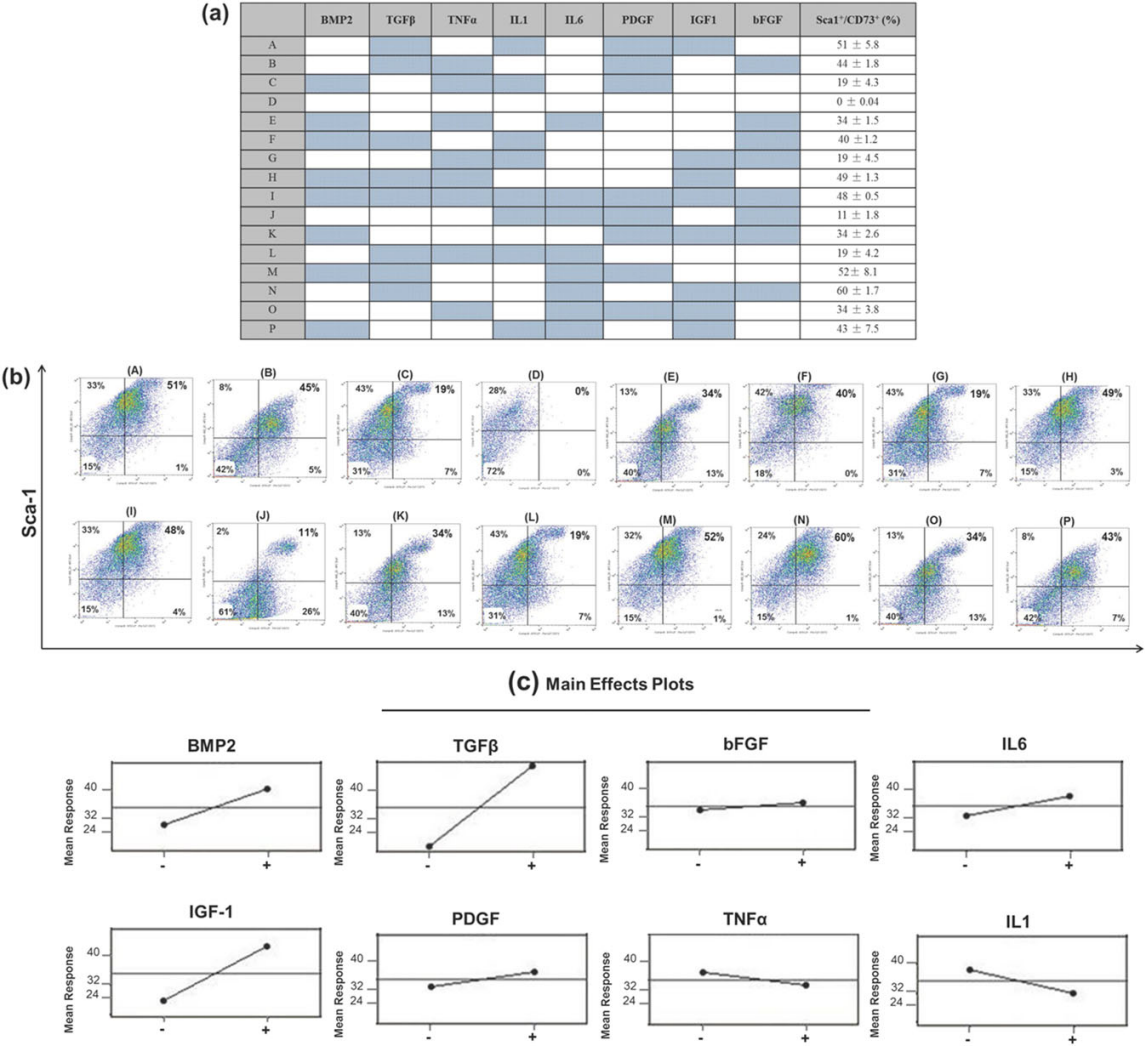
Principal component analysis (PCA) to determine main effects conducive to Sca‐1^+^/CD73^+^ phenotypic change. (a) Table and corresponding (b) flow cytometry data displaying the 16 different combinations of growth and inflammatory factors applied to bioengineered skeletal muscle cultures and the resulting percentage Sca‐1^+^/CD73^+^ positivity observed. Coloured cells signify the addition of growth/inflammatory factors. (c) Linear regression analysis of the main effects of each growth/inflammatory factor on the proportion of Sca‐1^+^/CD73^+^ cells. Flow cytometry data are represented as means ± standard deviation (*n* = 3). bFGF, basic fibroblast growth factor; BMP2, bone morphogenetic protein 2; IGF‐1, insulin‐like growth factor‐1; IL‐1/IL‐6, interleukin‐1/interleukin‐6; PDGF, platelet‐derived growth factor; TGFβ, transforming growth factor β; TNFα, tumour necrosis factor α

As the body's hyperinflammatory response to trauma is characterized by the presence of a large number of proteins associated with repair and chemotaxis, how combinations of these factors affected mineralization in the ‘reserve’ cell population was examined. To this end principal component analysis (PCA) was applied to examine the main effects of selected growth/inflammatory factors on conversion to a Sca‐1^+^/CD73^+^ phenotype (Figure 5). Results showed that a Sca‐1^+^/CD73^+^ phenotype was observed for all treatments (Figure 6a,b) excluding the untreated control (Figure 6b,d). Linear regression analysis of the main effects of each factor identified that BMP2 (*p* < 0.05), PDGF‐BB (*p* < 0.05), IL6 (*p* < 0.05) and bFGF (*p* < 0.05) significantly contributed to the generation of a Sca‐1^+^/CD73^+^ phenotype when added in the 16 combinations analysed (Figure 6c). Both TGF‐β and IGF‐1 caused the most significant (*p* < 0.005) increases in Sca‐1^+^/CD73^+^ phenotype, highlighting the possibility of indirect effects of these growth factors on mineralization within the ‘reserve cell’ population. In contrast, inclusion of TNFα or IL1 led to a significant reduction in the proportion of cells presenting with a Sca‐1^+^/CD73^+^ phenotype (Figure 6c).

**Figure 7:**
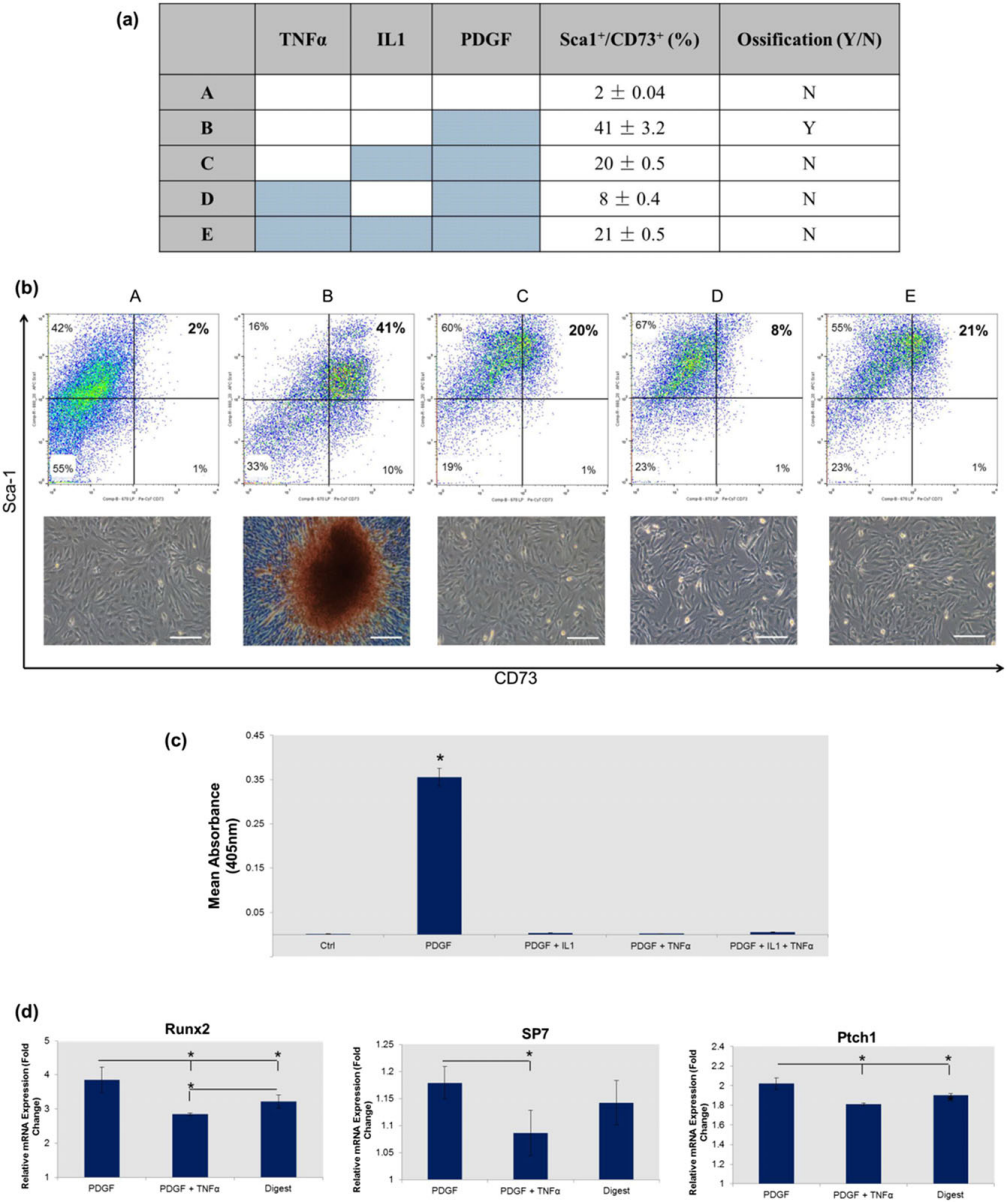
Pro‐inflammatory cytokines tumour necrosis factor (TNF)α and interleukin (IL)1 mediate platelet derived growth factor (PDGF)‐induced ectopic ossification in tissue‐engineered skeletal muscle. (a) Table displaying the combination of factors added to each culture and how this affected the proportion of Sca‐1^+^/CD73^+^ cells and the presence of ectopic mineral. (b) Flow cytometry data and Alizarin red staining showing the mediating effects of pro‐inflammatory cytokines on mineralization, and how this is related to the proportion of Sca‐1^+^/CD73^+^ cells. (c) Quantification of the concentration of Alizarin red stain bound in PDGF‐BB cultures exposed to pro‐inflammatory cytokines IL1 and TNFα. (d) Quantitative real‐time reverse‐transcription polymerase chain reaction gene expression analysis showing reductions in osteogenic regulators Runx2 and SP7, and the downstream hedgehog signalling factor Ptch1 in the presence of TNFα. Polymerase chain reaction was performed at day 3 with gene expression normalized to the untreated ‘reserve’ cells using the ΔΔCT (Livak) method. Coloured cells signify the addition of growth/inflammatory factors. Bars: 100 μm. *p < 0.05. Flow cytometry, polymerase chain reaction and Alizarin red quantification data are represented as mean ± standard deviation (*n* = 3)

Identification of a potential link between mineralization and an increase in Sca‐1^+^/CD73^+^ phenotype, together with the conclusions derived from PCA (Figure 6) led to examination of the potential modulatory effects of TNFα and IL1 in the presence of PDGF‐induced mineralization. Using flow cytometry profiling it was identified that the proportion of Sca‐1^+^/CD73^+^ cells formed following exposure to PDGF‐BB (41%) was significantly reduced in the presence of IL1 (19.7%, *p* < 0.05), TNFα/IL1 (20.5%, *p* < 0.05) and TNFα (8.64%, *p* < 0.005) (Figure 7a). Subsequent AR staining and quantification showed that mineralization induced by exposure to PDGF‐BB was prevented in the presence of all three conditions: TNFα, IL1 and TNFα/IL1 (Figure 7b,c).

As exposure to TNFα caused the most significant reduction in Sca‐1^+^/CD73^+^ phenotype, attention was focused on further characterizing the potential osteoinhibitory effects of this cytokine on PDGF‐BB osteoinduced cultures. A qRT‐RT‐PCR analysis identified a significant reduction in the expression of osteogenic regulators Runx2 and Sp7 in PDGF‐BB cultures exposed to TNFα (Figure 7d). The presence of TNFα in PDGF‐BB cultures also resulted in a significantly lower expression of the hedgehog signalling protein Patched 1 (Ptch1) (Figure 7d). Finally, PCR data showed that the addition of TNFα to PDGF‐BB cultures caused the expression of Runx2 to fall significantly below that of the untreated controls (Figure 7d).

## 4 Discussion

The C2C12 myoblasts in 3D culture contained both myogenic and undifferentiated ‘reserve’ cell populations. ‘Reserve’ C2C12s that had not differentiated to form myotubes exhibited limited myogenic potential after isolation from 3D gels and subsequent culture in myogenic medium. These findings contribute to data identifying heterogeneity within the C2C12 population (Deato and Tjian, 2007) and identify distinct similarities between ‘reserve’ cells and satellite cells (Chakkalakal *et al*., 2014). Further similarity is found when one considers that both C2C12s and satellite cells can be osteogenically induced in the presence of BMPs (Katagiri *et al*., 1994; Asakura *et al*., 2001). In particular, examination of genes expressed temporally during myogenesis identified similarities between ‘reserve’ cells and activated satellite cells, which are no longer quiescent but primed for differentiation. Much like activated satellite cells, ‘reserve’ cells downregulated their expression of the quiescence marker Pax7 in relation to the myogenic regulatory factor, MyoD1 (Stuelsatz *et al*., 2010). Expression of MyoD1 is often found to be reduced in cells committed to an osteogenic lineage (Ono *et al*., 2011). However, a study by Komaki *et al.* (2004) has shown that MyoD1 expression in satellite cells may also be responsible for cell plasticity, cooperating with Runx2‐activated osteocyte‐specific genes to coordinate osteogenic differentiation. Furthermore, knockdown of MyoD1 has previously been shown not to enhance satellite cell osteogenesis (Asakura *et al*., 2001), which suggests that this myogenic factor does not necessarily have an anti‐osteogenic role. Finally, comparatively low levels of the terminal differentiation factor myogenin, together with a lack of myoblast fusion, suggests that ‘reserve’ cells are likely to represent a population of activated muscle progenitors that have not been fully primed for myogenic differentiation, but may have a native or inducible osteogenic capacity.

Previous studies have highlighted the osteogenic potential of C2C12s (Katagiri *et al*., 1994). However, it appears that no previous reports have specifically demonstrated an osteogenic capacity within the ‘reserve’ cell population. The present study has shown that culturing C2C12s in a 3D collagen environment defines a method for the selection of a pre‐osteogenic ‘reserve’ cell population. These cells have been termed pre‐osteogenic because they demonstrated a comparative increase in the expression of the master osteogenic regulator Runx2 compared with unselected C2C12 controls, but no corresponding increase in SP7 or AR staining. These findings suggest that this population is primed for osteogenic differentiation, but that exogenous osteoinductive proteins are likely required for the transition to occur. Significantly, the addition of exogenous osteoinductive factors (BMP2 and PDGF‐BB) promoted mineralization in ‘reserve’ cell cultures, even in the absence of osteogenic medium with no differences seen in the unselected C2C12 controls. The fact that these cells were able to mineralize in standard growth medium without the addition of osteogenic medium suggests that ‘reserve’ cells were able to utilize limited exogenous sources of calcium and phosphate present within basic culture medium, such as calcium chloride (0.2 g/l) and sodium monophosphate (0.109 g/l), providing further evidence that these cells were primed for osteogenic differentiation. Previous studies have demonstrated the osteoinductive effects of BMP2 within skeletal muscle (Katagiri *et al*., 1994; Kaihara *et al*., 2004), and links are present between BMP‐induced ectopic bone formation and a rare genetic form of HO termed fibrodysplasia ossificans progressiva (FOP) (Shore *et al*., 2006). However, acquired HO may not be entirely BMP dependent, with a large number of factors possibly contributing to skeletal ossification at the wound site (Chromy *et al*., 2013).

We demonstrate that exposure to PDGF‐BB promoted significantly more mineral deposition and expression of key osteogenic markers in ‘reserve’ cell cultures than BMP2, the current gold‐standard osteoinductive factor in animal studies of HO. This cytokine is elevated as platelets infiltrate injured tissue following trauma (Liu *et al*., 2006) and a previous link between PDGF‐BB and ectopic ossification has been found in studies examining the mechanisms governing vascular calcification as a consequence of atherosclerosis (Boucher and Gotthardt, 2004). In addition, PDGF‐BB synthesis is elevated in areas of low oxygen tension via an association with hypoxia‐inducible factor‐1 (HIF‐1), a well‐established protein linked with HO (Schito *et al*., 2012). Perhaps the contribution of PDGF‐BB to HO has been most conclusively demonstrated by a study utilising imatinib, a PDGF‐BB antagonist, for the treatment of ectopic bone growth. The study showed that administration of imatinib significantly reduced the volume of ectopic bone formed in an Achilles tenotomy model of HO (Werner *et al*., 2013). However, it appears that no correlation between PDGF‐BB and skeletal muscle ossification has been documented previously. Unlike BMP2, PDGF‐mediated activation of osteogenesis does not occur via the Smad signalling pathway, which has been consistently implicated in hereditary forms of HO, such as FOP (Pignolo *et al*., 2011). It has been suggested that PDGF‐BB instead promotes osteogenic differentiation in MSCs through activation of the ERK1/2 MAPK pathway (Salasznyk *et al*., 2004), which has previously been identified as key pathway regulating osteoblast differentiation and skeletal development (Ge *et al*., 2007). However, further studies will be required to confirm the exact mechanism of PDGF‐BB on mineralization in the reserve cell population. A recent report on PDGF‐BB‐induced activation of Smad1/5/8 transcription factors via Erk5 further sheds light on crosstalk between MAPK and Smad signalling pathways (Tsioumpekou *et al*., 2016) and, together with the finding of the present study that exogenous PDGF‐BB promoted mineralization in the ‘reserve’ cell population, collectively it corroborates the importance of understanding the protein signalling cascade in the context of pathological ossification. Future work would involve verifying whether PDGF contribute to HO initiation via mechanisms involving latent response of traumatized muscle to overactive BMP signalling, as proposed by Shi *et al*. (2013).

**Figure 8:**
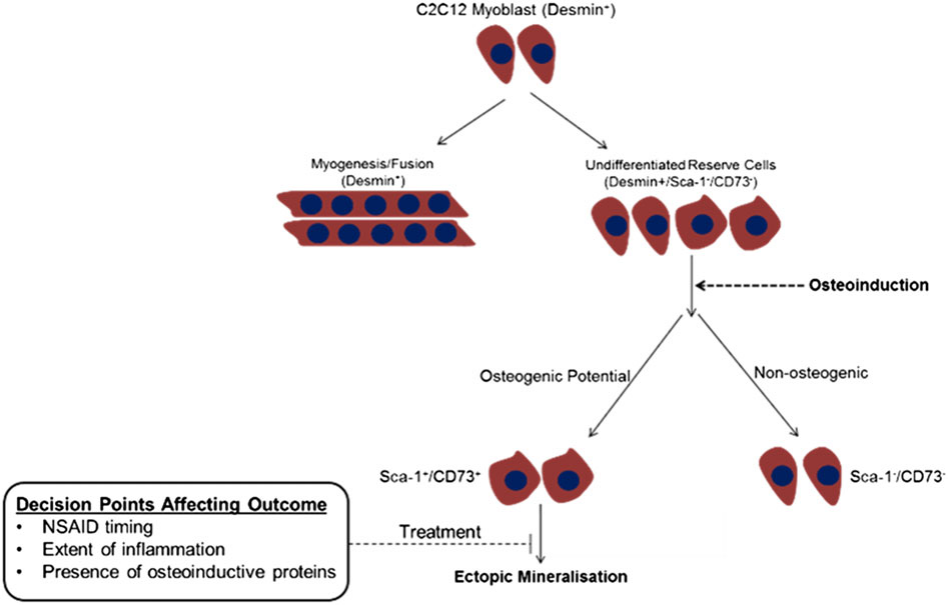
Diagram depicting a possible mechanism governing ectopic mineralisation in the tissue‐engineered skeletal muscle construct. C2C12 cells cultured in a three‐dimensional (3D) environment contain a population of cells that are able to fuse to form myotubes and a smaller population of cells that remain unfused, the so‐called ‘reserve’ cells. In the presence of osteoinductive factors, such as bone morphogenetic protein 2 (BMP2) or platelet derived growth factor (PDGF)‐BB, a proportion of these ‘reserve’ cells adopts a Sca‐1^+^/CD73^+^ phenotype. The phenotypically altered Sca‐1^+^/CD73^+^ population is osteogenic in nature and able to deposit a mineralized matrix. However, mineralization of the Sca‐1^+^/CD73^+^ population is mediated by the presence of pro‐inflammatory cytokines such as tumour necrosis factor α and interleukin 1. Hence, it is proposed that factors such as the level of inflammation, timing of non‐steroidal anti‐inflammatory drug (NSAID) administration, and relative abundance of osteoinductive proteins are likely to influence the outcome

Mineral deposition within the ‘reserve’ cell population appeared to correlate with the presence of a Sca‐1^+^/CD73^+^ phenotype, and the proposed mechanism by which these cells may contribute to ectopic mineralization in the skeletal muscle construct used in the present study is shown in Figure 8. Of particular note was the finding that growth factors causing only minor (TGF‐β) or no (IGF‐1) observable mineralization when added independently, were shown to facilitate the formation of a Sca‐1^+^/CD73^+^ phenotype when added in combination with other prominent factors linked with skeletal muscle trauma and HO (i.e. synergistic effects of TGF‐β and PDGF observed). This identifies a method by which factors secreted at the trauma site may indirectly contribute towards ectopic ossification, further highlighting the complexity of the association between post‐trauma inflammation and HO. The Sca‐1^+^/CD73^+^ cells were CD144^–^ and, as such, independent of previously identified non‐myogenic, pro‐osteogenic interstitial mesenchymal progenitor cells (Uezumi *et al*., 2010). These cells also did not express CD44 or CD90, thereby demonstrating there had been no reversion to a mesenchymal stem cell phenotype. Sca‐1 is expressed by myoblasts as well as stem and progenitor cells derived from a number of tissues, including muscle‐derived stem cells and myogenic precursors recruited to sites of injury. Although the exact function of this cell surface protein remains elusive previous studies have shown that Sca‐1 null mice present with osteoporosis, thereby identifying a link between Sca‐1^+^ cells and bone quality (Holmes *et al*., 2007). CD73 is a 5′ nucleotidase that converts ATP and AMP to adenosine, and has been shown to promote osteogenic differentiation of human mesenchymal stem cells and osteoblast differentiation (Takedachi *et al*., 2012). The present study provides evidence to suggest a link between a Sca‐1^+^/CD73^+^ phenotype and ectopic mineralization, and it is hypothesized that this may result from the reversion of ‘reserve’ cells to an osteoprogenitor‐like state. Previous studies have previously demonstrated dedifferentiation of terminally differentiated myoblasts (McGann *et al*., 2001; Chen *et al*., 2005). In addition, within the context of acquired HO, skeletal muscle cells have been shown to de‐differentiate into mononuclear cells capable of forming myoblasts, satellite cells or muscle‐derived stem cells in response to injury, thereby further establishing a link between myoblast dedifferentiation and HO (Mu *et al*., 2011). This is relevant given that acquired HO occurs in skeletal muscle as a consequence of trauma and suggests that myoblast reversion to a pro‐osteogenic cell type may be linked with the post‐trauma microenvironment. Furthermore, the correlation between CD73^+^ ‘reserve’ cells and *in vitro* mineralization, together with findings presented by Peterson *et al.* (2014) identifying that limiting extracellular ATP can inhibit bone formation at the site of HO (Peterson *et al*., 2014), implies that the presence of a pro‐osteogenic cell type is opportunistic and can be primed in response to a number of permissive microenvironmental factors.

Trauma is immediately followed by a hyper‐inflammatory response. In the present study it was shown that the presence of pro‐inflammatory cytokines, TNFα and IL1, can mediate PDGF‐induced mineralization. Tumour necrosis factor α is produced by activated macrophages during the post‐trauma inflammatory response, and the potential benefits of using TNFα to prevent endochondral ossification have been documented (Yoshikawa *et al*., 1988). Furthermore, TNFα has a prominent role in osteoclastogenesis, largely through its interaction with IL1 (Charatcharoenwitthaya *et al*., 2007). The finding that TNFα and IL1 blocked PDGF‐induced mineralization is highly pertinent because non‐steroidal anti‐inflammatory drugs (NSAIDs) are frequently prescribed as HO prophylactics (Beckmann *et al*., 2014). These drugs are often administered in combination with single‐dose radiotherapy to prevent HO formation following invasive surgeries such as total hip arthroplasty. However, reports concerning the efficacy of NSAID prophylaxis are mixed, and any association between inflammation and the development of HO complex. A recent study examining the mechanism by which preoperative radiation therapy acts to reduce HO showed that the number of immunological cells was significantly increased within the irradiated area, with a corresponding increase in pro‐inflammatory cytokines, possibly suggesting that inflammation is a negative regulator of HO (Hoff *et al*., 2013). The results of the present study, in conjunction with the other studies described further suggest that the presence of local inflammatory factors within skeletal muscle following trauma acts to prevent ossification within the myoblast population. These results consolidate existing studies questioning the efficacy of NSAIDs as a primary HO prophylactic, and suggest that the timing of NSAID administration is likely to be crucial for effective prevention of HO.

### 4.1 Limitations

Some of the limitations of the present study exist, highlighting the need for further research that will build on initial findings to fully clarify the role of PDGF‐BB and the immunological response during HO. First, it is acknowledged that the concentration of PDGF‐BB *in vivo* fluctuates widely over the course of HO development, with increases most likely to occur during platelet and immune cell aggregation. In the present study the concentration of PDGF applied was consistent with that reported in *in vitro* studies throughout the literature. However, it is acknowledged that there is a need for further study to examine the effects of concentration and timing of PDGF‐BB dosing in the system. An absence of immunological cells also restricts the number of conclusions that can be drawn in relation to the role of inflammation during HO, as the chemoattractive and activating effects of PDGF‐BB on these cells was outside the scope of the study. Lastly, because HO is a highly complex condition that is likely to result from the combinative effects of multiple cell types it is necessary to consider the effects of PDGF‐BB on local and migratory stem cells, endothelial cells and pericytes, which have all been implicated in this debilitating condition (Davies *et al*., 2015).

## 5 Conclusion

In the tissue‐engineered model used, PDGF‐BB was shown to be a potent inducer of skeletal muscle ossification. The presence of pro‐inflammatory cytokines, TGFβ and IL1, directly modulated the action of this osteoinductive factor on a population of undifferentiated ‘reserve’ cells, thereby providing a possible route for regulating HO and coordinating bone formation for regenerative applications. However, the processes governing HO are complex and the role of the post‐trauma inflammatory response is likely to be multifaceted. Therefore, the osteoregulatory effects of these cytokines will need to be tested in other contributory cell populations such as local and circulating osteogenic precursors, endothelial precursor cells and skeletal muscle progenitors (Davies *et al*., 2015). There is also evidence to suggest that the hyper‐inflammatory response to trauma may be in part genetically determined, suggesting that certain individuals are predisposed to developing HO after suffering a trauma.

## Conflict of interest

The authors have declared that there is no conflict of interest.
